# Nurses’ experiences and viewpoints about the benefits of adopting information technology in health care: a qualitative study in Iran

**DOI:** 10.1186/s12911-020-01260-5

**Published:** 2020-09-21

**Authors:** Jamileh Farokhzadian, Reza Khajouei, Arie Hasman, Leila Ahmadian

**Affiliations:** 1 Nursing Research Center, Kerman University of Medical Sciences, Kerman, Iran; 2 Medical Informatics Research Center, Institute for Futures Studies in Health, Kerman University of Medical Sciences, Kerman, Iran; 3 Department of Medical Informatics, Amsterdam UMC - Location AMC, Amsterdam, the Netherlands; 4 Department of Health Information Sciences, Faculty of Management and Medical Information Sciences, Kerman University of Medical Sciences, PO Box: 7616911313, Kerman, Iran

**Keywords:** Adoption, eHealth, Information technology, Nurses, Qualitative approach

## Abstract

**Background:**

Information technology (IT) plays an important role in nursing practice. Hence, nurses’ experiences and viewpoints about IT integration into healthcare help improve nurses’ adoption of IT. This study aimed to explore the nurses’ experiences and viewpoints about the benefits of IT integration and adoption in healthcare.

**Methods:**

This study was conducted with a qualitative research approach. Participants included 14 nurses from four hospitals affiliated to a large medical university in Iran, who were selected using a purposive sampling method. Data were collected through semi-structured interviews and analyzed using the conventional content analysis of Lundman and Graneheim.

**Results:**

Six categories in the study reflected the nurses’ experiences and viewpoints about the benefits of integrating IT into health care. These categories included improving the quality and efficiency of medical services and care, facilitating the communication management in the technological environment, improving information documentation, management, and monitoring, improving resource management, improving management performance and policymaking, and facilitating pathways of organizational and professional growth.

**Conclusions:**

Lessons learned in this study can help overcoming the barriers of IT adoption, and developing appropriate strategies to familiarize nurses with the benefits of IT in healthcare settings. Healthcare managers are recommended to investigate the experiences of nurses with IT in their hospitals and organize courses to orient hesitant nurses toward adopting IT.

## Background

Information technology (IT) has transformed healthcare and nursing in several aspects including clinical, management, education and research areas [1]. Nurses’ adoption of IT is critical to integrate IT successfully in the nursing fields [2]. Research conducted on IT adoption in healthcare has focused on two main areas. First, researchers extensively documented that introduction of IT into clinical settings, and nurses’ adoption of IT have positive effects or benefits for many nursing care aspects. For example, it has influenced time management of documentation and clinical activities, quality of documentation, nurses’ competencies and empowerment, and quality of care. Moreover, it improved intra- and inter-professional collaboration, nurse-patient relationship, teaching of patients and families,, and nurses and patients’ satisfaction [3].

Second, despite widespread efforts for facilitating IT adoption and presenting positive impacts or benefits of integrating IT into healthcare, several studies reported that nurses have not adopted such tools completely [4–7]. There are still some barriers (e.g. underutilization and reluctance to use IT, sabotage, nurses’ resistance to IT implementations, and unfavorable attitudes toward IT) to the successful interaction of nurses with IT in health care, and fitting IT into the workflow of nurses is challenging [6, 8]. The lack of IT adoption can result in unintended negative consequences such as anxiety, fear, a desire to return to paper system [9], deterioration of work conditions and quality, dissatisfaction, cognitive overload, exhaustion and sense of ineffectiveness [10]. Other reported consequences are the failure of IT implementations, or suboptimal user-system interactions, which in turn lead to nurses’ frustration, and adverse patient outcomes [11].

IT adoption can be reinforced by facilitating factors such as minimizing nurses’ discomfort about IT, exploring and understanding nurses’ experience about positive impacts and the benefits of integrating IT to their practice [6, 12]. On the other hand, the literature showed that IT adoption may vary greatly among different types of organizations and countries. It is also affected by cultural, socio-organizational factors, and demographic features such as hospital size, type of hospital (whether it is educational or not), ownership, location or area (urban or rural), budget, rate of IT staff, nurses’ awareness of the benefits of IT, and their IT competencies [13–15]. Therefore, qualitative studies presenting nurses’ experiences and viewpoints about the benefits of adoption of IT to diverse contexts will be worthwhile [16]. Moreover, most studies have used quantitative approach and investigated the impacts related to the adoption of IT in hospital settings. Few qualitative studies in various countries explored nurses’ experiences and viewpoints with IT implementation, facilitators and barriers to IT adoption. In the studies, the nurses mentioned both negative and positive experiences and viewpoints about IT adoption. Lack of training and awareness about the potential benefits of IT contributed to the negative experiences of the nurses. Researchers concluded that managers should refine policies to reflect benefits of IT in nursing practice. Previous studies recommended conducting further research to explore perceived benefits of IT adoption in different contexts [2, 17, 18].

The studies investigated barriers, facilitators, positive and negative impacts of IT adoption on health care. We found no comprehensive, qualitative study exploring the nurses’ experiences and viewpoints about the positive impacts or benefits of IT adoption in healthcare system of Iran. On the other hand, according to McKibbon, quantitative studies provide answers or insights for many important questions or issues in healthcare and clinical research. Other important questions dealing with why, how, contexts, and experiences of individuals or groups, can be best addressed using qualitative methods. Qualitative studies provide insight into social, emotional, and experiential aspects of healthcare [19].

IT has become a central part of nursing practice and using IT applications is one of the most important responsibilities of nurses [1]. Therefore, the objective of this study was to explore nurses’ experiences and viewpoints about the benefits of IT integration and adoption in hospitals in Iran using a qualitative approach. The lessons learned from this study, and sharing nurses’ experiences concerning IT adoption could be useful for other nurses.

## Methods

### Design and settings

This study was conducted using a conventional, qualitative, content analysis method. The content analysis approach is a method for systematic and rule-guided classification and description of text material considering words, phrases, latent contents and contexts [20].

The settings for this study were four teaching and referral hospitals affiliated with Kerman University of Medical Sciences located in Kerman. Kerman is the largest city in southeast Iran with 800,000 inhabitants. These hospitals receive patients from southeast of Iran, within the radius of 500 km. Different high-tech medical and surgical interventions are conducted in these hospitals by nursing and medical staff who collaborate with university-based medical scientists in educating healthcare students. Authorities of Kerman University of Medical Sciences select appropriate e-Health applications for the hospitals. These hospitals have more than 20 years of experiences of using IT and are among IT pioneers in Iran. All healthcare policies implemented in hospitals are centrally made by Iranian Ministry of Health. The political agenda is currently paying more attention to application of e-Health, and all hospitals are actively implementing Hospital Information System (HIS). HIS is used to automate hospital tasks such as cost management, resource management, medication management, office automation, accounting, nurses and physicians’ performance assessment, and patients’ registration.

The distribution of nurses is similar in all the hospitals throughout Iran. The majority of nurses have bachelor’s degrees and the recruitment of nurses follows the same pattern in all the hospitals. Nurses in the organizational chart of the hospitals are classified into five main positions including matron or nurse manager, clinical and educational supervisors, head nurse, and nurse or clinical nurse. Usually nurses rotate among different clinical wards according to the decision of the matron. Hence, all nurses involved in our study had at least experience of working in more than one clinical ward, No registered nurse (RN) and informatics nurse specialist are currently working in the study settings. There are currently no policies in place addressing nursing informatics competencies for nurses. Normally, after the implementation of a HIS, nurses receive both brief medical informatics and regular computer training. Also a short training is provided in the wards when new functionalities are released. Each hospital has an IT department. IT staff working in the IT departments of the hospitals have enough knowledge of all aspects of e-Health applications and guide the nurses during the Implementation of the HIS. HIS in-service training is currently done in clinical wards for the new nurses.

### Participants and sampling

A purposive sampling method was applied, and 14 nurses were selected to participate in this study (Table 1). In each hospital, the first researcher, with assistance of nurse managers, selected a number of eligible participants. She asked the nurse managers to suggest nurses who would be representative of their peers and to speak with openness. The sampling process was continued based on the principle of maximum variation to capture rich and diverse perspectives and experiences of the participants. Therefore, nurses with different genders, ages, years of work experience, degrees, positions, and clinical wards were enrolled until data saturation. All of the selected interviewees had several years of work experiences in their professions. They were normally working with IT such as a HIS and most of them have been working with paper-based systems for several years.

**Table 1:** Demographic information of participants

Participant	Gender	age-ranges	Position	Work experience-ranges	Degree
1	F	37–46	Head nurse	11–20	Master
2	F	37–46	Head nurse	11–20	Master
3	F	37–46	Clinical nurse	11–20	Bachelor
4	M	37–46	Clinical nurse	2–10	Bachelor
5	M	37–46	Clinical nurse	11–20	Bachelor
6	M	27–36	Clinical nurse	2–10	Bachelor
7	F	27–36	Clinical nurse	2–10	Bachelor
8	M	27–36	Clinical nurse	2–10	Bachelor
9	F	47–55	Clinical nurse	21–30	Bachelor
10	M	47–55	Clinical nurse	21–30	Bachelor
11	F	37–46	Officer of quality improvement	21–30	Master
12	F	47–55	Educational supervisor	21–30	Master
13	F	37–46	Educational supervisor	21–30	Master
14	F	37–46	Clinical supervisor	21–30	Bachelor

### Data collection

Data collection was performed through open-ended semi-structured interviews. The first researcher, a PhD nurse, who had a background in clinical nursing and experienced in-depth interview for qualitative studies, conducted all the interviews. She went to different wards, explained the study objectives to nurses, invited them to participate in the study, and scheduled an interview date. All interviews were conducted face-to-face, audio recorded and transcribed. Topic-guide questions were developed based on specific study objectives through consensus of the research group who were faculty members of nursing and medical informatics. Initially, a series of stimulus questions were designed and asked to create a friendly atmosphere and to prompt responses of the participants in the qualitative techniques. Then, the interview questions sought nurses’ experiences and viewpoints about the benefits of IT adoption in healthcare. Some of the guide questions are as follows: what are the benefits of IT adoption in healthcare or in nursing practice? Does healthcare and nursing practice improve when using IT? How? Do you think that IT has changed healthcare? If yes, please explain the changes due to the use of IT compared with the paper-based system. Then the interviewer asked follow-up probing questions to clarify relevant avenues of inquiry raised by the participants and to elicit nurses’ experience and deeper narrations, such as “please explain more about …”.

Interviews with nurses were conducted in their hospitals after receiving their consent, and they were organized in a way not to disrupt daily schedules of the participants. The interviews took 40–65 min in a quiet room near the ward or another location in hospitals based on the participants’ preference.

### Data analysis

The data were analyzed based on instructions proposed by Lundman and Graneheim [21]. In the first step, every interview was transcribed verbatim by the first researcher. In the next step, two researchers (JF, LA) read the transcriptions several times to obtain an overall understanding of the content, determine meaning units, perform an initial coding and interpret the data individually. The two researchers manually merged codes and classified them based on their similarities, and established sub-categories and categories independently. Then, they developed the coding scheme (code name, code definition, categories, subcategories, and text examples, and coding rules). In this process, the researchers met regularly to discuss about agreements and discrepancies of assigned codes, categories, subcategories, and rationale. Coding disagreements > 5% were discussed by the research team to reach consensus and the coding scheme was revised. Initial agreement on coding was high (92%), and the researchers discussed their interpretations to further refine concepts and resolve disagreements until achieving a consensus. Finally, all researchers reviewed coding scheme and triangulated them which formed the final coding scheme. At each consensus meeting, to assess coding agreement, we calculated the percentage of agreement for the most frequently coded sections of the transcripts. Coding agreement and inter-coder reliability were obtained when both coders assigned the same code to the main idea of a segment from a transcript. The overall inter-coder reliability for the transcript was high (91%) [20]. Moreover, two external experts specialized in qualitative research reviewed and approved the coding process and categories. Coding and data analysis were conducted using MAXQDA10 (qualitative data analysis software), which enhanced the comprehensibility of the analytic process.

The trustworthiness of the data was determined using Lincoln and Guba’ criteria, including credibility, confirmability, dependability, and transferability [21]. To ensure these criteria, the researchers were constantly working on the data, were in contact with the participants for 1 year, and established a friendly relationship with the participants. Furthermore, summaries of the interviews and results were presented to participants, and they were asked to provide feedback on the findings. The co-researchers continuously reviewed and verified the coding and categorization methods in frequent meetings and the findings were assessed in the context of prior literature. In addition, we presented our findings to the nurse manager of each hospital and used their comments for the interpretation of the findings.

## Results

### Demographic information

The participants included two head nurses, eight clinical nurses, two educational supervisors, a clinical supervisor, and a quality improvement officer. The nurses were nine women and five men aged 27–55 years with work experiences ranging from 2 to 30 years. Five nurses had a master’s degree and the others had a bachelor’s degree in nursing (Table 1).

### Results from interviews

In the analysis of the 14 interviews, six categories and 21 subcategories emerged (Table 2). Results along with quotations presented by participants are described in the following.

**Table 2:** The categories and subcategories extracted from data

Categories	Subcategories
1. Improving the quality and efficiency of medical services and care	-Reducing clinical risks and error
	-Increasing staff safety
	-Improving decision-making efficiency
	-Maintaining continuity of care
	-Improving workflow (work process) and planning care
2. Facilitating the communication management in the technological environment	-Improving inter-organizational communications
	-Improving and expanding the intra-organizational communications
3.Improving information documentation, management, and monitoring	-Benefitting from simultaneous documentation and care
	-Increasing data and documentation accessibility
	-Increasing integrity of information
	-Creating a precise and secure archive of documents and information
4. Improving resource management	-Reducing workload and paper work
	-Saving non-nursing staff
	- optimizing and purposeful activities
	-Saving time and costs
5.Improving management performance and policy making	-Reinforcing a dynamic and responsive structure
	-Improving accreditation and audit
	-Facilitating the staff’ performance evaluation
6.Facilitating pathways of organizational and professional growth	-Creating aspiration to improve the informatics competencies
	-Enhancing virtual training and facilitating the training process
	-Developing technology according to the needs of healthcare providers

#### Improving the quality and efficiency of medical services and care

##### Reducing clinical risks and error

According to the nurses, the care provided through IT is more accurate and have a higher quality and safety. IT gives nurses a sense of control over the patients’ condition and reduces clinical risks and errors.

*“When the patient*’*s blood group is recorded as O*^*+*^ *in the HIS, errors of the laboratory staff are largely prevented.” (p 12)*

Application of IT also has decreased medication errors and improved drug management, and created an integrated medical error reporting system.

*“When an error occurs, we fill in the online error reporting forms. The error analysis authority shares online corrective error strategies with all wards. Before this system, nurses wasted a lot of time on writing paper forms, now the forms are in the computer which make reporting and analyzing much easier.” (p3)*

##### Increasing staff safety

Regarding access to integrated information of the patients’ status provided by HIS, nurses have a feeling of confidence and peace of mind. They have control over the situation, because all documentations are available and these documents cannot be manipulated or deleted from the HIS.

*“A CT scan was made in the emergency ward, but it was lost in the surgery ward. The IT department determined that the CT scan was removed from the patient profile in the internal medicine ward; so, the personnel of surgery and emergency wards were acquitted.” (p1)*

##### Improving decision-making efficiency

IT plays a major role in providing the required and valid information for the decision-making process of the healthcare team. For example, application of IT can reduce the time needed to transfer information to the healthcare team and decisions are made timely with a maximum efficiency.

*“The patient was not feeling well and we informed the physician about the patient’s condition directly and the necessary measures were taken within an appropriate time. Documentation with HIS can facilitate this process within a few minutes.” (p10)*

##### Maintaining continuity of care

One of the most basic responsibilities of health care team is to maintain continuity of care. IT leads to continuity of care, for example, with providing quick access of physicians to the results of diagnostic and laboratory tests and graphs. Therefore, time is saved and the treatment and care process is not delayed. Another benefit of IT is protection of patients’ data during the delivery of shifts, acceleration of shifts’ delivery, and reduction of delays in the care process at the time of shift change.

*“Physicians can easily enter the patient*’*s national code at the clinic and observe a CT scan or X-ray report. They can diagnose the disease and start the medications very quickly.” (p2)*

##### Improving workflow (work process) and planning care

Rapid access to patients’ information such as the results of para-clinical test accelerates the work process and improves the nursing care and treatment plans. Finally, the health care team’s satisfaction increase.

*“At night, the physician observed the X-ray results of a patient in her office using the Picture Archiving and Communication System (PACS) and started the medication procedures immediately.” (p2)*

*“With the help of HIS, the patients*’ *discharge process is facilitated, and we prepare the bed for the next patient rapidly.” (p.9)*

#### Facilitating the communication management in the technological environment

##### Improving inter-organizational communications

Accurate communication and timely transmission of information are necessary in healthcare settings. Information confirmation and reception is easy and effective when it is done simultaneously between care teams in different departments of the hospital. For example, electronic health records (EHR) have created a wide communicational system between therapeutic wards and para-clinical units such as pharmacy, radiology, support, revenue, admission, and discharge. Furthermore, IT facilitated communication of care team with patients and increased their trust on the care team.

*“I fill out the equipment breakdown form in the HIS; the equipment authority sees it in his system simultaneously and comes to fix it fast. Moreover, all the personnel in the ward know about the damaged equipment and the time of their fixation.” (p5)*

##### *Improving and* expanding the *intra-organizational communications*

Nurses explained that health information of individuals is stored in an EHR throughout their lives. Therefore, in case of a treatment requirement anywhere in the country, the individual’s profile will be accessible through a computer network using the national code. In addition, IT has facilitated transfer of information to the Ministry of Health and among other hospitals and healthcare organizations.

*“When a pregnant mother*’*s death scenario is entered into the national maternal deaths system from a hospital, the Ministry of Health immediately investigates the case and makes plans at the macro level to reduce maternal deaths. Then, the related instructions are sent to all the hospitals of the country.” (p 12)*

#### Improving information documentation, management, and monitoring

##### Benefitting from simultaneous documentation and care

Today, simultaneous documentation with care help nurses record care events on time. In the same vein, IT help provide careful and comprehensive care, reduce the time required to document reports, and increase the available time to provide direct care to patients.

*“The patients*’ *history in EHR helps the triage; when the patient has a cardiovascular disease, I will prioritize him/her and inform the physician. I also take emergency measures such as tests.” (p2)*

Nurses pointed out that access to a comprehensive set of information about patients could speed up nursing process. However, the nursing process and nursing classifications such as nursing diagnoses, the nursing interventions classification (NIC) and the nursing outcomes classification (NOC) which is applied internationally for documentation of nursing records, and care plans are still paper-based in the study settings. They expressed a desire to take benefit of IT and have the classifications in electronic format in HIS.

##### Increasing data and documentation accessibility

If the data are not available in a timely manner, their capture will be useless. All data should be readily accessible at the time of need for clinical, administrative, and organizational purposes. Further benefits of IT include speeding up access to previous records of patients, increasing the accuracy of patients’ data registration, increasing the accuracy of hospital data, receiving daily statistics and reports, and reporting hospital income and expenses.

*“When we want to see the changes in the hemoglobin levels of the patient with bleeding from the time of admission, we simply open the system and compare the test results. However, if we wanted to search the paper files, the test results would be unavailable, lost, or unordered.” (p1)*

##### Increasing integrity of information

Registration of all information in the HIS and recording all care processes have ensured the integrity and uniformity of the information. This information can be used for treatment, research, and planning. Many of these data, such as nosocomial infections and adverse events are sent to the Ministry by electronic systems.

*“HIS provides a rich network of information for the hospital to conduct research, and managers can calculate a lot of indicators using this information.” (p 13)*

##### Creating a precise and secure archive of documents and information

Participants also pointed out that IT has created a convenient and secure archive for documents, so that information and documentation will never be lost.

*“The paper files may be torn or destroyed by events such as fire. However, it is easier to find documents and view statistics in the electronic archive, and there is no risk of missing information because the IT department often makes backups of information.” (p11)*

#### Improving resource management

##### Reducing workload and paper work

Participants highlighted the important benefits of IT in reducing paperwork and staff’s job load as well as saving paper consumption.

*“In paper work, we used to spend much time to keep the documents without folding papers. For example, we had to send a large number of paper requests for blood of the patients of cesarean section daily. Many of these requests were returned because of errors and thus turned into waste. All these are now electronic and such problems are no more available.” (p14, p1)*

##### Saving non-nursing staff

As the findings show, IT saves non-nursing human resources. For example, it reduces staff movement among wards. Previously, most non-nursing personnel were constantly moving between wards to deliver documents. Today, however, information is easily exchanged with IT and most non-nursing personnel tasks are done by IT.

*“We used to regularly send nursing staff to take test request with the samples for example the laboratory requests, but now we can see the results quickly in the HIS.” (p10)*

##### Optimizing and purposeful activities

Application of IT has reduced many repeated and unnecessary tests and measures. As a result, the burden on family caregivers has reduced.

*“Formerly, a lot of patients*’ *X-rays were disposed or archived at a great cost. However, the PACS saves both patients’ and hospitals costs. Calculation of expenses is much easier and no service record is eliminated or thrown away”. (p2)*

Nurses also believed that with IT, many targeted measures were carried out without time delay and cost.

*“Our social workers open the HIS in their room and check which patients need help. So, every day they go to the target patient purposefully.” (p11)*

##### Saving time and costs

Nurses described IT as a facilitative factor in rendering care services, speeding up tasks, and saving costs and time for patients and nurses. In addition, IT provides the opportunity for nurses to spend more time with patients.

*“Previously, when we wanted to get information from head nurses or sent a letter to them, an individual should deliver letters or collect responses from all wards. This process was very long and time consuming. Now, head nurses can see the letter at the same time in HIS.” (p11 and p12)*

#### Improving management performance and policy making

##### *Reinforcing a dynamic and responsive* structure

Enough information and statistics are beneficial for senior managers to make timely, logical, and specialized decisions and policies. IT has led to timely detection of shortcomings, fast access to financial and organizational reports, accurate reporting and accountability to executives, and appropriate communication with financial managers for mid-level and operational executives. Moreover, IT helps control performance and strengthens accountability and responsiveness of employees, since records and documents are firmly recorded for all actions with date and time.

*“The HIS is an audit system. So, staff should pay attention not to lose sheets of paper in the patient's profile and physicians are committed to protect the graphs and services they carry out, because no service can be eliminated, and they must be responsive.”) p3)*

##### Improving accreditation and audit

IT has facilitated quality improvement actions such as auditing and accreditation through the inter-organizational communication network. All data concerning the implementation of work processes, policies, indicators, and reports of patient safety events are recorded and monitored. Then, the relevant documents are used for accreditation and audit purposes.

*“We have created a folder called “Accreditation Standards” in the HIS for all departments and shared all accreditation actions in this folder. Personnel review this folder, complete the forms related to accreditation actions, and send them to us.” (p11)*

##### Facilitating the staff’ performance evaluation

Managers need valid information to control, monitor, and evaluate personnel’s duties, which are facilitated by IT. Moreover, staff’s self-assessment and participation in the process of performance evaluation is mentioned as another benefit of IT.

*“All staff should upload and submit their self-assessment documents in an electronic evaluation system. Finally, staff’s performance evaluated by the nurse manager is observable in electronic profiles.” (p13)*

#### Facilitating pathways of organizational and professional growth

##### Creating aspiration to improve the informatics competencies

Nurses believed that with combination of technological advances in nursing practice, they must acquire and update their informatics competencies in various ways, such as learning from colleagues, participating in training courses inside and outside the organization, and self-directed learning.

*“I became more enthusiastic about updating my informatics competencies. I tried to attend educational courses inside and outside the hospital. If I had any problems, I would ask my colleagues or the IT department staff in the hospital or group discussion sessions” (p4)*

##### Enhancing virtual training and facilitating the training process

IT boosts training in medical professions in both virtual and classroom learning. For example, electronic exchange of patients’ files between different treatment centers, discussions about diagnosis and treatment of patients, and application of patients’ information such as results of X-rays at training sessions help the individual and professional development of the health care team. Moreover, by application of the virtual learning system, personnel are not always required to attend the classes.

*“In one ward, a patient had a blood transfusion problem; the novice nurse did not know what to do. I told him, on the phone, to refer to the blood transfusion folder, read the instructions how to manage blood transfusion problems, and to take the necessary action.*” *(p14)*

Participants also believed that the process of training and completing personnel training records is much faster and less costly with IT.

*“I enter the educational program in the system each month and the personnel sign up for the classes they need. At the end of each class, they will receive certificates.” (p13)*

##### Developing technology according to the needs of healthcare providers

The deficiencies of IT will be detected while nurses are using it. Nurses and nurse managers have an effective role in determining deficiencies when developing IT by conducting periodic reviews to detect problems in healthcare because of deficiencies in IT.

*“In the previous version of HIS, some codes, such as bronchoscopy of lung parenchyma, were not defined for our ward. However, the new version added these capabilities. Gradually, defects were corrected and patients and nurses are more comfortable.” (p5)*

## Discussion

The present study explored nurses’ experiences and viewpoints about the benefits of IT integration and adoption in healthcare. The results depicted a picture of various beneficial outcomes of IT in nursing practice classified into six categories. One of the categories was “improving the quality and efficiency of medical services and care”. The findings of our study are in agreement with previous studies on IT adoption and implementation in a variety of settings. For example, our results have confirmed the findings of a systematic review that aimed to evaluate the influence of eHealth on nursing care. This review study reported that the use of IT by nurses can affect their practice, modify the ways in which they plan, provide, document, and review clinical care and finally improve the quality of care provided and patients outcomes [3]. Other studies have also reported that IT adoption increases the quality of patient care and improves the standards in healthcare organizations [6] by supporting clinicians in making clinical decisions efficiently [22–24]. Moreover, it reduces the risk of making errors by decreasing the clinicians’ cognitive workload, synthesizing and organizing information in accessible and usable formats [25]. IT improves providers’ performance by reducing delays in care at the time of shift change and maintains continuity of care [23].

Our study participants frequently expressed development of an integrated medical error reporting system as an important beneficial outcome of IT adoption. These systems are considered among essential factors for implementing patient safety plans and also the most important steps to learn from errors and previous experiences. This issue was not examined adequately in the previous studies. Unlike our results that IT improves patient outcomes, there are controversies about benefits of IT in the studies [24, 26]. Varghese et al. reported problems and unintended consequences of IT implementation such as errors and delays in clinical decision-making, and actual or potential patient harm because of technical and sociotechnical or context factors [24].

In the present study, “facilitating the communication management in the technological environment” along with its subcategories was identified as another perceived benefit for IT. Similarly, two studies maintained that IT improved communication between/among wards and staff [23, 27], teamwork and team support [25]. A systematic review on this topic reported several themes, including the impact of EHR on information exchange between patients and clinicians; maintaining positive relationships, and partnering with patients; and the EHR as an amplifier of existing communication behaviors [28]. Another systematic review explained that IT had both positive and negative impacts on communication. Negative impacts were as follow: clinicians should divide their attention by the patient and computer, had difficulty in maintaining eye contact, and other aspects of patient-centeredness. This literature review suggests that by applying appropriate strategies, IT can support patient-clinician interaction rather than interfere with it. These positive impacts included improving nurses’ communication skills, increasing information sharing between patients and clinicians and increasing opportunities to engage with and to empower patients by sharing computer-based information and resources with them and providing opportunities for patients to ask questions and providing more information to the clinician. These positive impacts depends to a large extent, on nurses’ styles of interacting with IT and patients as well as how they adapt to use IT in the workflow [7]. In our study, participants emphasized on theme of expanding the intra-organizational communications, which was less reported in the mentioned review articles. We recommend that nurses use IT for the patient care by adopting some of the strategies such as learning the verbal and nonverbal cues to support their interactions with patients. For this purpose, educators can develop interventions and educate nurses how to integrate IT into clinical settings.

Another perceived benefit for integration of IT in healthcare system was improving information documentation, management, and monitoring along with its subcategories. The findings of our study support a previous systematic review that evaluated the impact of information and communication technologies on nursing care [3]. Another study also showed that IT could manage patients’ information, increase the legibility of recorded data, increase patients’ security of information and documents, increase data accessibility, and improve accuracy of information and activities [23]. Moreover, Saranto et al. explained that IT has facilitated the use of international nursing classification such as nursing diagnoses, NIC and NOC, which have been translated into various languages for providing patient care and documentation of the nursing process. Nursing classifications as a standardized nursing language (SNL) have been developed to describe the nursing process, to document nursing care and to facilitate aggregation of data for comparisons at the local, regional, national and international levels. Therefore, these classifications should be linked to one other in HER. Researcher reported that many countries transferred paper-based to electronic documentation and used SNL in electronic nursing records [29]. In our study, nurses were concerned about using present information systems or electronic nursing records that do not support SNL nursing classifications. Nurses suggested that SNL should be created and linked to one other in HIS to track nurses’ contributions to patient care electronically.

“Improving resource management” with subcategories was another perceived benefit for integration of IT in healthcare. These results were in agreement with previous studies that reported the benefits of IT adoption such as reduced treatment costs and resources, reduced task time and increased working speed, faster transmission of lab test orders, reduced staff movement between wards, reduced paper work and consumption, reduced workload, improved review of patients’ records to plan their care, improved exchange of information, increased hospital revenue [23]. Other studies also showed that the data collected with HIS are clear, accessible, complete, timely, and relevant [30], reduce burden on family caregivers [31], and repeated imaging [32]. In other words, healthcare professionals can use these data to provide high-quality services, and managers are able to manage their resources, including staff and tools, more efficiently [30]. The IT benefited nurses by helping them perform their daily tasks with greater ease, and it made the nurses’ work more effective*.* IT improved nurses’ efficiency by reducing resource consumption and facilitating information access, recording, and processing [33].

Another category emerged from interviews was “improving management performance and policy-making” with subcategories. These results were in agreement with studies that reported nurses’ adoption of HIS had the potential to influence on policy-making and management of nurses who used such technologies at work [16]. Similarly, empirical studies of nurses’ IT adoption showed that IT contributed to improving accreditation, audit, management performance [23], increasing accountability, and responsibility [34] .Another research showed that IT was very advantageous for quality control, quality assurance, process monitoring, and process optimization programs [30]. Rahimipour et al. noted that IT is an important part of the staff empowerment process and improvement of human resource capabilities. In addition, IT increases staff’s knowledge, creativity, and quality of performance [35].

The other category extracted in this study was “facilitating pathways of organizational and professional growth” with subcategories. In this regard, previous studies reported that enhancing informatics competencies are important for the acceptance of HIS and similar technologies [16, 36]. In addition, researchers emphasized that IT plays an important role in education and nursing work. IT has created opportunities for utilization of e-learning and education, improvement of training services, public education, patient and their families’ education. IT has increased access to resources and educational content at any time and place, saved time and cost and created more opportunities to learn different ways and improve the quality of education and standard of nurses training [1, 17, 37]. Another study found that the adoption of IT by healthcare providers contributed to gradual system evolution [34], improved EHR design [25], and stimulated users to add useful functions to the system [38]. In a study by Zakane et al. nurses argued that the application of a clinical decision support system increased their knowledge, and these systems could be used as continuous training for nurses [39]. Blank et al. also showed the positive impacts of nursing decision support system on the improvement of nurses’ skills and knowledge [40]. According to our findings, nurse mangers provide opportunities for nurses and use multiple educational pathways to equip them with required competencies for IT adoption to nursing practice. Moreover, schools of nursing must prepare students for IT adoption through competency-based approaches, reexamination and updating nursing curricula based on new IT advances, and changes in the needs of patients and the healthcare system.

In total, the results of this study may be somewhat similar to other studies. However, there are the discrepancy between some of our results and the above-mentioned studies. These can be due to the differences in community, sampling study design, technical and socio-organizational or context factors. For example, in our study, a qualitative design was used to explore the deep experiences of nurses from the benefits of IT adoption. We suggest that future studies employ a standard approach to examine the effect of these sociotechnical contextual variables, as well as system, human and other key factors on the IT adoption and their contribution to positive outcomes such as quality and safety.

### Limitations

The study had several limitations. First, this study explored benefits of IT adoption based on the experiences of nurses working in the healthcare system of Iran, and it is difficult to generalize the findings to all professional groups or other context. Other benefits may be perceived in different cultural and organizational contexts, with other groups, e.g., physicians, and even from a multidisciplinary perspective. Second, this study did not focus on negative experiences or viewpoints of nurses about IT adoption. However, the nurses mentioned negative impacts of IT during the interviews that were not within the scope of this study. Future studies should endeavor to overcome the mentioned shortcomings associated with this study. In this regard, we suggest conducting further studies exploring positive and negative impacts of IT adoption in different cultural and organizational contexts, with other groups. Third, we did not compare nurses’ perceptions of paper and electronic system; quantitative studies can supplement a qualitative study to explore the benefits of integrating IT compared with using paper systems. Such comparative studies will increase the reliability of the results and will help researchers achieve comprehensive results and enrich insights.

## Conclusions

Understanding of nurses’ experiences and viewpoints about benefits of IT and lessons learned in this study can contribute to the IT adoption, overcome the barriers, and develop appropriate strategies to orient nurses towards the benefits of IT adoption in healthcare settings. The results suggest that the healthcare managers invite nurses from the studied hospitals to share their positive experiences and use them to develop appropriate strategies and policies for improving IT adoption among nurses and other medical professions. When nurses are hesitant or have a negative feeling, managers can organize courses for orientating a positive direction. Furthermore, researchers can explore factors that enhance nurses’ adoption of new IT in the research area and other contexts. Probably, providing users with positive feedback, technical support, and good training programs can help designers facilitate IT adoption. Regarding the expansion and specialization of nursing, nursing informatics experts should be recruited to promote the services provided by nurses in educational, research, clinical, and managerial areas.

## Data Availability

The data analyzed in this study is not included in a repository because it contains interview transcripts.

## Abbreviations

IT: Information technology
PACS: Picture Archiving and Communication System
HIS: Hospital Information System
HER: Electronic health records
SNL: Standardized nursing language
NIC: Nursing interventions classification
NOC: Nursing outcomes classification
